# A Comparative Study of Voice Characteristics in Children With Cochlear Implants and Typically Hearing Children: Insights From an Indian Context

**DOI:** 10.7759/cureus.48050

**Published:** 2023-10-31

**Authors:** Akshat Kushwaha, Arun Alexander, Anuprasad Sreenivasan

**Affiliations:** 1 Otolaryngology, Jawaharlal Institute of Postgraduate Medical Education and Research, Puducherry, IND; 2 Otorhinolaryngology, Jawaharlal Institute of Postgraduate Medical Education and Research, Puducherry, IND

**Keywords:** speech perturbation, bilateral profound deafness, voice parameters, cochlear implantation, congenital deafness

## Abstract

Purpose

The aim of the study was to evaluate speech outcomes in children with cochlear implants compared to normally hearing children in terms of fundamental frequency, shimmer, and jitter. The study also aims to assess the intelligibility of speech in children with cochlear implants using a speech intelligibility rating scale.

Methods

This was a hospital-based comparative study conducted at JIPMER, a major tertiary referral center. A total of 25 prelingually deaf children with profound deafness, who underwent cochlear implantation at the institute, were recruited from the outpatient department of the Department of Otorhinolaryngology. Twenty-five children under seven years of age who underwent cochlear implantation and received a minimum of 36 speech therapy sessions were included in the study. Subjects with incomplete electrode array insertion and any neurological maldevelopment were excluded. Age- and gender-matched controls comprising 25 individuals were selected from the Ophthalmology Outpatient Department at JIPMER.

Study procedure

The study commenced in January 2019. Test subjects were asked to visit the Audiology and Speech and Language Pathology Department at JIPMER. Voice recordings were conducted in a soundproof room using a microphone, with the mouthpiece held at a distance of 10-15 cm from the patient. The patient was instructed to say "a" three times. Their voice was recorded and analyzed using Praat software (Version 6.1.15, developed by Paul Boersma and David Weenink, Phonetic Sciences, University of Amsterdam). Data were analyzed using IBM SPSS Statistics for Windows, Version 19 (Released 2010; IBM Corp., Armonk, New York) (Department of Biostatistics, Vanderbilt University, Nashville, Tennessee, USA), and the results were derived.

Results

The mean fundamental frequency for Group 1 (CI) was 266.03 ± 57.46 Hz, compared to 312.97 ± 22.15 Hz for Group 2 (NH). There was a statistically significant difference between the values of both groups, indicating that cochlear implantation positively impacted the fundamental frequency of speech. The study revealed a significant change in the fundamental frequency when children were implanted at an early age and received effective speech therapy post-implantation. This change was assessed after one year post-implant. Perturbation measures such as shimmer and jitter were lower in the cochlear implant group but were not statistically significant.

Conclusion

Children with congenital bilateral severe to profound sensorineural hearing loss tend to have higher values of the fundamental frequency of speech. However, when implanted at an early age, they showed a significant difference in the fundamental frequency of speech (p < 0.001). Speech perturbation was lower in the post-cochlear implant group, with a statistically significant difference in the values of shimmer alone. The study concludes that children with cochlear implants can achieve normal voice parameters with early intervention and training. However, the variability range is much higher than in typically hearing individuals.

## Introduction

The way a person speaks forms a fundamental element of the initial impression they create, as well as serving as a means of effectively conveying information. The qualities of one's speech impact how their thoughts are expressed and communicated to others, potentially making them more appealing, engaging, or intellectually stimulating [1].

Although there is a scarcity of data elaborating on the role of auditory cues in shaping an individual's speech outcomes, existing literature indicates that there are significant variations in certain vocal parameters in individuals with hearing loss compared to those with normal hearing [2]. This is due to the vital role that auditory feedback plays in influencing both individual speech elements, such as word or syllable selection, as well as broader aspects of speech and voice production, including tone, pitch, and loudness. It is also crucial for monitoring second-to-second, real-time segmental features of speech [3,4]. Hearing loss leads to faulty voice production, which can cause social, psychological, and speech limitations, thus affecting an individual's overall quality of life.

Severe to profound hearing loss affects both speech reception and production skills, especially in cases of prelingual deafness, where these skills have not been developed at all. Thus, even after mitigation of deafness, these individuals may require sustained audiological training and speech therapy sessions to catch up with their normal-hearing peers.

The loss of auditory feedback leads to voice and speech perturbations [5]. Studies provide sufficient evidence that voice characteristics of individuals with hearing impairment differ considerably from those of individuals with normal hearing.

A cochlear implant is a device that bridges this gap and provides the auditory perception and, thus, the feedback required for effective speech production. It is a novel, sophisticated neuro-prosthetic device that enables direct stimulation of the auditory nerve, even in the absence of a functional middle and inner ear hearing mechanism.

With advancements in technology and the advent of multichannel implants, the transmission of more spectral information to the auditory nerve is now possible. This has helped in improving sound reception using these devices. Even then, speech outcomes remain unpredictable in these individuals. This is due to variable and faulty auditory feedback, as cochlear implant speech processing systems find it extremely difficult to analyze the dynamic range of speech and deliver its fine temporal and spectral information that normal-hearing listeners use [6,7].

It is still unclear what a child with an implant is actually hearing and how it differs from that of normal individuals. The effect of this altered auditory feedback on the patient's speech remains poorly understood. However, available literature suggests that children with cochlear implants may have difficulties controlling the pitch and loudness of their voices during vocalization, and this variation is due to faulty auditory feedback [8].

The present study aims to examine the variations in speech outcomes of implanted children, in an attempt to understand the delicate interplay responsible for these variations. Enhancing our understanding of this pivotal interaction will facilitate the improvement of auditory feedback design for children with cochlear implants, with the ultimate goal of replicating the auditory experience of individuals with typical hearing. To achieve a level of auditory feedback that mimics a normal or near-normal experience, it becomes essential to assess the disparity in vocal parameters between implanted children and their peers with typical hearing.

Objectives

The objectives of this study are to evaluate speech outcomes in children with cochlear implants as compared to children with normal hearing with regard to fundamental frequency, shimmer, and jitter. Additionally, the study aims to assess the intelligibility of speech in children with cochlear implants using the Speech Intelligibility Rating Scale designed by O'Donoghue et al. in 1999 [9].

## Materials and methods

Study design and study setting

This study was a hospital-based comparative analysis conducted at a tertiary referral center. A total of 25 prelingually deaf children with profound deafness, who underwent cochlear implantation at the institute, were recruited from the outpatient clinic of the Department of Otorhinolaryngology. Ethical approval was obtained from the Human Ethics Committee of the Institute under the identifier JIP/IEC/2018/278. Written informed consent was also obtained from the guardians of all study subjects. The study adhered to the principles of ethical research as outlined in the Declaration of Helsinki, first established in 1975 and revised in 2008, as well as the Indian Council of Medical Research Guidelines for Biomedical Research on Human Participants.

Study Participants

The criteria for enrolling study participants are detailed in Table 1.

**Table 1:** Inclusion and exclusion criteria for study participants

S. No.	Inclusion criteria	Exclusion criteria
1	All children below 7 years of age who underwent cochlear implantation.	Incomplete electrode array insertion
2	Children with at least 1 year of post-implant period.	Children with any neurological maldevelopment
3	Received a minimum of 36 speech therapy sessions.	

Sampling Population

The sampling population consisted of two groups. Group 1 included children with bilateral profound sensorineural hearing loss who underwent cochlear implantation and were seen in our Otorhinolaryngology outpatient clinic. Group 2 consisted of individuals with normal hearing, who were matched in age and gender to the study participants. These individuals were selected from the ophthalmology outpatient department, as these children had no significant medical conditions that could impact their auditory perception in any manner.

Sampling Method

Consecutive sampling was performed.

Sample Size Calculation

In a previous study by Srividya et al. [10], it was found that the mean shimmer value was 3.35 dB with a standard deviation of 1.26. The response within each subject group was normally distributed. To be able to reject the null hypothesis that the population means of the experimental and control groups are equal with a probability (power) of 0.8, we needed to study 25 test subjects and 25 control subjects. The Type I error probability associated with this test of the null hypothesis is 0.05. We selected this specific study for comparison because it closely resembled our current research; both investigations were conducted in the southern region of India. Furthermore, they shared similarities in terms of post-implantation speech therapy rehabilitation conducted in regional languages, which could potentially influence the findings of our study.

Study procedure

Subjects were chosen as per the aforementioned selection criteria. Children with cochlear implantation (Advanced Bionics (AB) cochlear implants (HiRes 90K Advantage implant with straight and slim J electrodes) with a minimum of 36 speech therapy sessions) were asked to visit the Audiology and Speech and Language Pathology Department, of the institute where each speech therapy session consisted of two-hour audio-visual training in the regional Tamil language. Voice recording was done in a soundproof room using a microphone with the mouthpiece held at a distance of 15cm. The patient was asked to say “A” three times. Their voice was recorded and analyzed using the Pratt software (Version 6.1.15, developed by Paul Boersma and David Weenink, Phonetic Sciences, University of Amsterdam). Acoustic parameters were obtained by processing the recorded voice with a default application in the software.

The intelligibility of speech in children with cochlear implants was assessed in the same sitting by using the speech intelligibility rating scale as depicted in Table 2.

**Table 2:** Speech intelligibility rating scale Designed by O’Donoghue et al. [9]

Category	Criteria
5	Connected speech is intelligible to all listeners. The child is understood easily in an everyday context.
4	Connected speech is intelligible to a listener who has little experience of a deaf person’s speech.
3	Connected speech is intelligible to a listener who concentrates and lip-reads.
2	Connected speech is intelligible. Intelligible speech is developing in single words when in context and lip-reading cues are available.
1	Connected speech is unintelligible. Pre-recognizable words in spoken language; the primary mode of communication may be manual.

The demographic parameters of the patients were collected and recorded in the data collection proforma during the same sitting.

Statistical analysis

Data were analyzed using IBM SPSS Statistics for Windows, Version 19 (Released 2010; IBM Corp., Armonk, New York), provided by the Department of Biostatistics at Vanderbilt University in Nashville, Tennessee, USA, and results were subsequently derived. All statistical tests were considered significant at a p-value of less than or equal to 0.05. The normality of the data was evaluated using the Kolmogorov-Smirnov Test of Normality. For normally distributed data, statistical analyses for mean, standard deviation, and Student's t-test were performed to ascertain the statistical significance between the two groups. Mann-Whitney U test was used for non-normally distributed data.

## Results

Out of the 25 test subjects in Group 1, 22 (88%) were male and 3 (12%) were female. The subjects ranged in age from 3.5 to 7 years, with a mean age of 5.16 ± 0.93 years. This group was compared with an age- and gender-matched control group, designated as Group 2, which had a mean age of 5.14 ± 0.99 years. Both groups were found to be comparable using the Independent Samples T-test, with a p-value of 0.9361.

The normality of the data was evaluated using the Kolmogorov-Smirnov test of normality, and the resulting p-values are presented in Table 3.

**Table 3:** Table showing p-values calculated by the Kolmogorov-Smirnov test of normality CI: cochlear implant group, NH: normal hearing group.

Sl. No.	Acoustic parameter	Group 1 (CI)	Group 2 (NH)
1	Fundamental frequency (Hz)	0.79322	0.46608
2	Jitter (%)	0.04043	0.33383
3	Shimmer (Db)	0.00005	0.61366

As evident from Table 3, the fundamental frequency of speech (Hz) recorded was normally distributed (as p > 0.05) and, the mean with standard deviation was calculated. Jitter (%) and Shimmer (dB), on the other hand, were not normally distributed (p < 0.05), thus the median with inter-quartile range and p-values between both the groups were calculated (Table 4).

**Table 4:** Fundamental frequency, shimmer, jitter, and p-values of the two groups The p-value for fundamental frequency between both the groups is <0.001, showing a significant statistical difference implying that cochlear implantation has a positive impact on the fundamental frequency of speech. A similar positive effect is seen for the voice perturbation parameter Shimmer but not for jitter where there is no significant statistical difference between both the groups S: statistically significant, NS: statistically not significant

Sl. no.	Acoustic parameter	Group 1	Group 2	p-value
1	Fundamental frequency (Hz), mean ± SD	266.03 ± 57.46	312.97 ± 22.15	<0.001 (S)
2	Jitter (%), median-IOR	1.90 (1.44-4.44)	1.91 (1.63-2.70)	0.977 (NS)
3	Shimmer (dB), median-IQR	1.20 (0.77-1.73)	2.02 (1.66-2.40)	0.001 (S)

In cochlear implantees, the intelligibility of speech is a measure of how their speech is comprehended by others in a given condition. It is a measure of how these kids can interact with their peers. In the study, the intelligibility of speech was assessed using the Speech Intelligibility Rating Scale (SIRS). This number of implantees in each subgroup is given in Table 5.

**Table 5:** Speech intelligibility of implantees recorded on the SIRS scale Speech intelligibility rating scale (designed by O’Donoghue et al. [9])

SIRS category	Criteria	Number (%)
5	Connected speech is intelligible to all listeners. The child is understood easily in an everyday context.	3 (12%)
4	Connected speech is intelligible to a listener who has little experience of a deaf person’s speech.	11 (44%)
3	Connected speech is intelligible to a listener who concentrates and lip-reads.	9 (36%)
2	Connected speech is intelligible. Intelligible speech is developing in single words when in context and lip-reading cues are available.	2 (8%)
1	Connected speech is unintelligible. Pre-recognizable words in spoken language; the primary mode of communication may be manual.	0 (0%)

As evident from Table 5, 80% of the test subjects were SIRS categories 4 and 3, implying the positive impact of cochlear implantation where these kids were able to interact with their normal hearing peers with intelligible speech. 

## Discussion

Mean fundamental frequency of voice discussion

Among the various options possible, bilateral severe to profound sensorineural hearing loss (>91dBHL) is best mitigated by cochlear implantation. In children with congenital, bilateral, severe to profound sensorineural hearing loss, an early operation is preferred as neural pathways are maximally plastic until 3.5 years of age. 

In this study, the prelingually deaf pediatric population was studied. After the cochlear implant surgery, the children were asked to come at four weeks during the postoperative period to link the inner device with the speech processor. During this point, the child hears the sound for the first time. Speech training was subsequently initiated and children with a minimum of 36 speech therapy sessions were assessed for their acoustic parameters like fundamental frequency (f0) and perturbance parameters like shimmer and jitter.

We studied two groups of 25 candidates each, of which 22 candidates were male and 3 female. Group 1 included patients who underwent cochlear implantation and Group 2 comprised of age and gender-matched controls. 

The literature suggests that due to poor auditory feedback, the children with profound or severe hearing loss have difficulty in controlling their pitch but children with cochlear implants after a period of speech training tend to have control over their pitch resulting in normal fundamental frequency range, but the range seems to be wider [3,10,11]

The present study observed that the mean fundamental frequency (MF0) of Group 1 (CI) is lower than that of Group 2 (NH) for both genders. The mean fundamental frequency of Group 1 (CI) was 266.03 ± 57.46 Hz as compared to 312.97 ± 22.15 Hz of Group 2 (NH) candidates. Although it is in the normal range, there is a statistically significant difference in the values of both groups, indicating that the cochlear implant had an impact on the fundamental frequency of the test subjects.

Srividya et al. [10] compared voice parameters in 30 cochlear implant users and showed similar results with a statistically significant difference in the fundamental frequency of speech between both the groups. Group 1 with cochlear implant had a lower value of fundamental frequency (277.26 ± 47.88 Hz) as compared to normally hearing candidates (322.60 ± 60.19 Hz) in Group 2 (NH). This study differed from our study in terms of larger sample size and additional parameters studied such as amplitude perturbation quotient (APQ), voice turbulence index (VTI), and soft phonation index (SPI). 

The present study and the above study support the view that earlier intervention and training helps children with hearing loss achieve good auditory feedback and control on their pitch, which is reflected in the reduction of mean fundamental frequency post-implantation.

Another study done by Upadhyay et al. [11] on 42 prelingually deaf cochlear implant users showed higher and statistically significant difference (p < 0.001) in values of mean fundamental frequency in the implant group (313.67 ± 35.28 Hz) in comparison with the control group (289.95 ± 19.13 Hz). This difference could be attributed to the lower age of implantees in this study (2.97 years) as compared to our study as speech therapies post-implantation and the compliance of cochlear implant users for those therapy sessions might be poor in younger implantees. This study differed from our study in terms of a larger sample size. They also did a subjective analysis of voice comprising of aerodynamic and auditory perception evaluation by assessing maximum phonation time and GRBAS score respectively. Our study data extrapolated with the data of the two studies are shown in Table 6. 

**Table 6:** Fundamental frequency of speech of present study population in comparison to the reference studies by Srividya et al. [10] and Upadhyay et al. [11]

Fundamental frequency	Srividya et al. [10]	Upadhyay et al. [11]	Present study
Group 1 (CI)	277.26 ± 47.88 Hz	313.67 ± 35.28 Hz	266.03 ± 57.46 Hz
Group 2 (NH)	322.60 ± 60.19 Hz	289.95 ± 19.13 Hz	312.97 ± 22.15 Hz
Statistical difference	p<0.05	p<0.001	p<0.001

From Table 6, it is evident that the fundamental frequency of speech in our study population (266.03 ± 57.46 Hz) showed less deviance from the normal values as compared to the other two studies. The literature suggests that children with congenital hearing loss due to their poor auditory feedback have difficulty in controlling pitch. However, if implanted early in age, restoration of auditory feedback enables these children to control and monitor their pitch resulting in lower values of the fundamental frequency of speech. Our study also supports the view that earlier intervention and training help children with hearing loss achieve good auditory feedback and control of their pitch. This helps them attain near-normal auditory feedback via the cochlear implant and helps build a speech similar to their normal hearing peers. 

In another study, Hocevar-Boltezar et al. [2] showed a statistically significant fall in fundamental frequency measurements of thirty-one cochlear implant users who were implanted after an age of four years, where the mean fundamental frequency 288.19 ± 67.53 Hz before cochlear implantation improved to 259.38 ± 60.98 Hz (p = 0.018) within a period of six months post-implantation, thus strengthening the evidence that earlier implantation and proper speech training post-implantation can give a near normal speech to a child with congenital deafness. The study differed from ours in that they studied vocal parameters before cochlear implantation and subsequently at 6, 12, and 24 months post-implantation. Also, an additional parameter of HNR (harmonic-to-noise ratio in dB) was studied. A gender-wise analysis revealed better outcomes in female children than in males. It is indicated in the literature that girls with cochlear implants have been found to show higher scores than boys on tests of speech perception [12] speech production [13], language [14], and reading [15,16]. In our present study, girls have shown a better outcome in terms of control of fundamental frequency where the value is 239.486 Hz as compared to 269.65 Hz for boys. Since the number of female participants was very limited in our study, statistical tests are likely to be erroneous and any statistical significance is difficult to prove.

Voice perturbation parameter discussion

Vocal perturbation parameters such as Jitter and Shimmer indicate the stability of the phonatory system. The present study observed lower values of perturbation parameters in patients with a cochlear implant. The jitter and shimmer values in our study population were 1.91% and 1.20 dB, respectively. 

This can be explained by poor neuromuscular control of laryngeal muscles due to still-developing but poor auditory feedback. The current study showed lower values of jitter and shimmer in the cochlear implant group.

Srividya et al. [10] showed higher values of perturbation parameters in the CI group. The mean frequency perturbation parameters showed that the values are though statistically insignificant (except for Jitter percentage in the female category) but are higher than normal values. The mean intensity perturbation parameter is observed to be statistically significant both in males and females. 

Upadhyay et al. [11] showed higher values of perturbation measures in the cochlear implantation group and the difference was found to be statistically significant (p<0.05). The mean Jitter% of the cochlear implantation group was 0.65% and the normal hearing group was 0.39%. The mean shimmer (dB) of the cochlear implant group was 2.74 dB and the other group was 1.65 dB.

A study done by Bolfan-Stosic and Simunjak [16] measured Jitter and Shimmer values in hearing-impaired children and were significantly elevated compared to normal. Coelho [17] found higher values of jitter and shimmer in the children with cochlear implants, while in Baudonck’s study [18], the values of these parameters were higher in normal-hearing children. The data of the above studies have been extrapolated with the data of our study and are presented in Table 7.

**Table 7:** Comparison between jitter and shimmer of the present study with other reference studies CI: cochlear implant, NH: normal hearing, S: statistically significant, NS: statistically not significant

	Jitter (CI)	Jitter (NH)	p-value	Shimmer (CI)	Shimmer (NH)	p-value
Our study	1.90 % (1.44%-4.44%)	1.91 %± 0.62 %	0.977 (NS)	1.20 (0.77-1.73) dB	2.08 dB ± 0.59 dB	0.001 (S)
Srividya et al. [10]	1.78 % ± 1.36 %	1.38 %± 0.58 %	0.143 (NS)	0.33 dB ± 0.09 dB	0.46 dB ± 0.14 dB	<0.001 (S)
Upadhyay et al. [11]	0.65 % ± 0.44 %	0.39 % ± 0.13 %	<0.001 (S)	2.74 dB ± 1.04 dB	1.65 dB ± 0.40 dB	<0.001 (S)
Coelho et al. [19]	1.32 %	1.24 %	-	3.73 dB	3.35 dB	-
Baudonck et al. [18]	1.19 % ± 0.66 %	1.84 % ± 0.83 %	0.000 (S)	3.62 dB ± 2.04 dB	3.71 dB ± 1.32 dB	0.089

It is clear from the above table that the perturbation values were variable in different studies. However, the current study is in agreement with that of Baudonck et al. [18], where both jitter and shimmer are lower in the cochlear implantation group. This suggests a positive impact of cochlear implantation on the control of speech perturbations. In contrast, Upadhyay et al. [11] and Coelho et al. [19] showed higher values of both parameters in the cochlear implantation group. Srividya et al. [10] demonstrated a higher value of jitter but significantly lower values for shimmer in the cochlear implant group. 

The perturbation parameters, shimmer and jitter, are lower in the cochlear implantation group, and the range of values is within normal limits in our study. This suggests a positive outcome of implantation on speech and language rehabilitation. However, since serial data were not measured in our study, an objective assessment of improvement could not be made.

Although the difference in shimmer value between both groups is statistically significant, the actual difference is only 0.88 dB, which is a meager difference in voice intensity between implanted and non-implanted children. Similarly, frequency perturbation, jitter, in cochlear implant children is similar to that in children with normal hearing. Thus, shimmer and jitter may not be significant in representing the deviation of voice in the implant group in a real-world scenario. However, it is crucial to emphasize that these speech perturbations have a far-reaching impact on a child's overall quality of life. They directly influence the cognitive development and social skills of hearing-impaired children, playing a pivotal role in their ability to establish positive relationships, forge friendships, and engage in learning through active listening, speaking, and inquiring. It is important to acknowledge the following limitations of our study when discussing the study's results and conclusions.

Training and Socioeconomic Factors

The need for dedicated training of children to develop speech is a valid concern. It is noted that most patients in our study come from economically disadvantaged backgrounds, which could impact the availability and effectiveness of speech therapy. This socioeconomic factor could introduce bias and affect the generalizability of our findings.

Lack of Objective Analysis

The absence of an objective analysis for speech intelligibility, such as a connected speech test, is another limitation. Objective measures are crucial for a more robust assessment of the study's outcomes. Without such measures, the assessment of speech quality may be subjective and less reliable.

Cochlear Implant Usage Duration

Our study did not account for the duration of cochlear implant usage on voice quality. This could be an important factor, as the long-term effects of cochlear implants on speech quality might differ from short-term effects. Not considering this factor could limit the comprehensiveness of our findings.

Serial Assessment

The study did not include a serial assessment of vocal parameters to monitor improvement and changes in speech quality over time for individual subjects. This omission could make it challenging to track progress and the effectiveness of interventions.

Hence, it would be prudent for future research endeavors to take into account these constraints. This would offer a more thorough and dependable understanding of the influence of cochlear implants on speech quality in children, particularly those from underprivileged backgrounds. Additionally, future studies should focus on uncovering the direct social consequences of hearing loss and effective management strategies. To comprehensively grasp the intricate relationship between hearing impairment, speech development, and strategies for enhancing speech outcomes, a more holistic approach may be necessary to positively impact the personal and social development of hearing-impaired children.

## Conclusions

The study revealed differences in the values of the fundamental frequency of speech and voice perturbation measures among children with a cochlear implant compared to children with normal hearing. Children with congenital bilateral severe to profound sensorineural hearing loss tend to have higher values of the fundamental frequency of speech. However, when implanted at an early age, they showed a significant difference in the fundamental frequency of speech (p < 0.001).

Speech perturbation parameters such as shimmer and jitter were found to be lower in the group of children who had received cochlear implants. There was a statistically significant difference in the values of shimmer specifically. Nevertheless, it is crucial to recognize that these parameters play a pivotal role in the social and personal development of these children, directly influencing their ability to contribute to society.

The study's conclusion highlights that, with early intervention and training, children with cochlear implants can attain voice parameters within the normal range. However, it is worth noting that the variability in these parameters is much broader in this group compared to individuals with typical hearing.

This study underscores the profound impact of hearing loss on the social, psychological, and emotional development of children and emphasizes the importance of early diagnosis and intervention for congenital hearing loss. Such early intervention is crucial for ensuring that these children can lead fulfilling lives and actively participate in society.

To gain a comprehensive understanding of the intricate relationship between hearing loss and psychosocial development, future research endeavors may be necessary. Such research could lead to the development of novel strategies aimed at optimizing the quality of life for individuals affected by hearing loss.
